# Vulgar Psoriasis Triggered by Active Pulmonary Tuberculosis: A Case Report and Literature Review Highlighting Immunological Interactions and Genetic Susceptibility

**DOI:** 10.3390/clinpract15040071

**Published:** 2025-03-28

**Authors:** Alexandra-Cristiana Gache, Alexandra-Florentina Bîlbă, Andreea-Raluca Pricop, Elena Danteș

**Affiliations:** 1 Faculty of Medicine, ‘Ovidius’ University of Constanta, 1 University Alley, 900470 Constanta, Romania; andreearaluca24@yahoo.com (A.-R.P.); elena.dantes@gmail.com (E.D.); 2 County Emergency Clinical Hospital, 145 Tomis Boulevard, 900591 Constanta, Romania; 3 Dermatology Clinic of County Emergency Clinical Hospital, 126 Ștefan Cel Mare Street, 900178 Constanta, Romania; dr.alexandrabilba@yahoo.com; 4 Clinical Hospital of Pneumophtisiology, 40 Sentinelei Street, 900002 Constanta, Romania

**Keywords:** tuberculosis, psoriasis, TNF-α, Apremilast, immunomodulation

## Abstract

**Introduction:** About one in four people show an immunological reaction to an infection with *Mycobacterium tuberculosis*, which can remain latent or lead to active forms of the disease. Psoriasis is a chronic, immune-mediated skin disease that can be associated with numerous comorbidities. Biologic therapies have revolutionized psoriasis treatment but carry the risk of reactivating latent tuberculosis infection. However, the link between tuberculosis as a triggering factor for the onset of psoriasis remains unknown. **Clinical Case:** We present the case of a patient initially diagnosed with secondary pulmonary tuberculosis, who, two months after the diagnosis, showed a remarkable clinical evolution by developing lesions consistent with vulgar psoriasis, necessitating a multidisciplinary treatment approach. **Discussions:** This unique case highlights the shared immune mechanism of these diseases, particularly involving TNF-α, IL-17, and CD4+ T cells. **Conclusions:** The coexistence of these conditions raises critical questions about the interplay between infectious and autoimmune diseases and the impact of genetic susceptibility.

## 1. Introduction

Tuberculosis (TB) poses a significant threat to global public health because, although it is a preventable and treatable disease, it is the 13th leading cause of death worldwide and the second most important infectious agent after the coronavirus responsible for COVID-19 [1]. About one in four people worldwide show an immunological response to infection with *Mycobacterium tuberculosis* (Mtb), which can remain latent or progress to active forms of the disease [2].

Psoriasis is a chronic, immune-mediated inflammatory skin disease that is associated with numerous comorbidities, including psoriatic arthritis, mental disorders, and cardiovascular and liver diseases. This condition affects both sexes; however, it was observed to present at an earlier age in women and individuals with a familial predisposition [3]. The age onset of psoriasis is bimodally distributed, with the incidence being highest in men aged 30–39 years and 60–69 years in women [4]. According to the World Health Organization, psoriasis was classified as a severe non-transmissible disease in 2014, also highlighting the suffering caused by misdiagnosis, inadequate treatment, and the stigma associated with this condition [5].

In recent years, biological therapies have revolutionized the treatment of immune-mediated inflammatory diseases, including psoriasis. However, there is evidence that specific biological therapies used in psoriasis, particularly those based on tumor necrosis factor (TNF) inhibitors, can reactivate latent infection with Mtb. Therefore, in the clinical treatment of psoriasis, it is essential to screen for TB infection before initiating biological therapy [6].

Nevertheless, the role of TB in the development of psoriasis remains uncertain, as there is currently very little data on this topic in the specialist literature. Understanding these mechanisms could help to explore new pathogenic pathways and optimize diagnostic and therapeutic strategies.

This article aims to analyze the possible common immunological mechanisms of TB and psoriasis and compare these observations with existing data in the literature by presenting this clinical case and highlighting the clinical implications of this phenomenon.

## 2. Detailed Case Presentation

### 2.1. Clinical Findings

A 27-year-old male patient, a non-smoker with no occupational exposure to pollutants and no significant personal medical history, was admitted to the Constanța Clinical Hospital of Pulmonology with symptoms characterized by persistent dry cough, febrile episodes at home, and pleuritic chest pain.

The family history states that five years ago, the patient’s father was found to have latent TB infection, during routine screening, prior to initiating biologic therapy for psoriatic arthritis. The father subsequently underwent chemoprofilaxy before starting immunosuppresive treatment.

No significant abnormalities were found on admission during the general physical examination. Upon inspection, the patient exhibited a mildly affected general condition, with a normal BMI of 20.4 kg/m^2^ and no psoriasiform lesions or other cutaneous eruptions. Hemodynamically stable and with preserved cardiorespiratory function, the patient showed no evidence of superficial palpable lymphadenopathy. Pulmonary auscultation revealed vesicular breath sounds present bilaterally but with decreased intensity at the right lung base.

### 2.2. Blood Workup, Bacteriological Exams, Radiological Findings, and Bronhoscopy

Blood tests showed a moderate inflammatory syndrome characterized by leukocytosis with monocytosis and low uric acid levels (Table 1).

**Figure 1:**
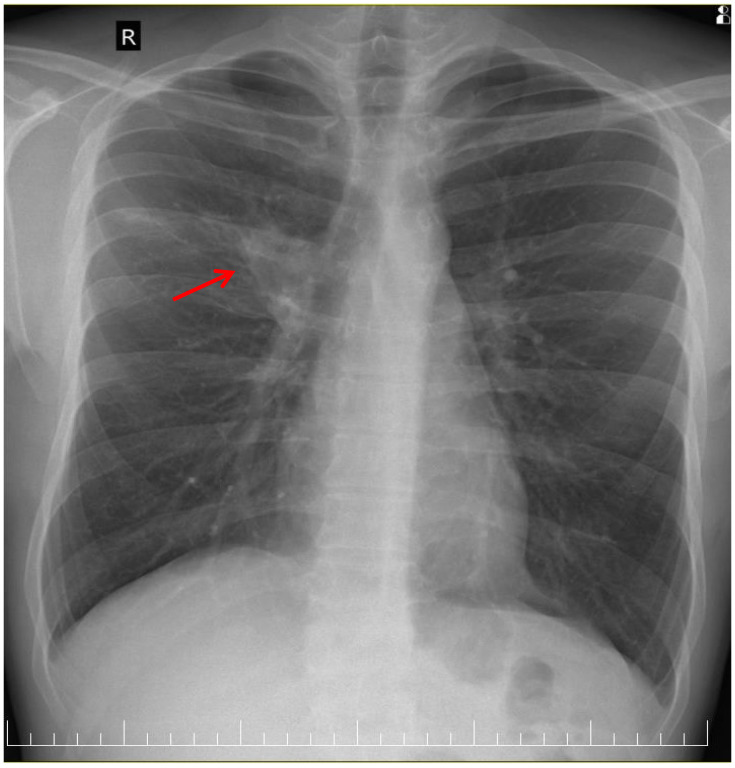
Chest X-ray showing high-intensity opacity in right suprahilar region (red arrow).

**Figure 2:**
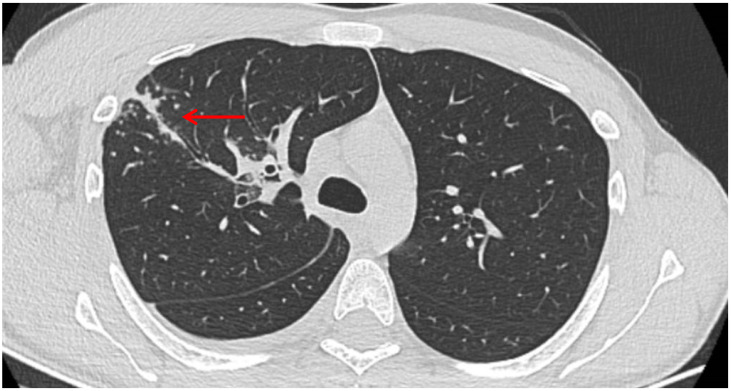
Lesions that showed a characteristic “tree-in-bud” pattern in the right upper lobe, along with fluid accumulation in the right pleural cavity (red arrow).

A posteroanterior chest radiography (chest X-ray) before admission revealed a high-intensity opacity with indistinct margins and a heterogeneous appearance, localized in the right suprahilar region (Figure 1). Subsequent native and contrast-enhanced chest computed tomography scans (CT) identified centrilobular micronodules and consolidations associated with linear lesions that showed a characteristic “tree-in-bud” pattern in the right upper lobe, along with fluid accumulation in the right pleural cavity, suggestive of secondary pulmonary TB or other infectious ethiology (Figure 2).

Initially, sputum cultures for bacterial flora on blood agar, chocolate agar, and Grigalski media, as well as fungal culture on Sabouraud medium, were performed, all yielding negative results.

In parallel, tuberculosis screening was initiated, including the Mantoux test (IDR to 5 U PPD) and Quantiferon testing, both of which returned positive. However, it is important to note that these tests do not distinguish between latent tuberculosis infection (LTBI) and active disease. Additionally, the patient underwent sputum examination for Mtb, which was also negative on microscopy.

Due to persistent clinical suspicion, fibro bronchoscopy was conducted. It revealed a normal laryngopharynx, trachea, and carina, with cough hyperreactivity and no evidence of bronchial stenosis or proliferative lesions. Bronchoalveolar lavage was carried out, and the collected samples were submitted for laboratory analysis.

### 2.3. Diagnosis, Treatment, and Monitoring

Taking into account the clinical presentation (fever, night sweats, persistent cough) and radiological findings, one of the initial differential diagnoses considered was atypical virobacterial pneumonia. However, HIV testing was negative, ruling out an underlying immunodeficiency as a contributing factor. As a result, empirical antibiotic therapy was initiated with a third-generation Cephalosporin intravenous (Ceftriaxone 1 g every 12 h/day) alongside antitussive treatment. After three days of antibiotherapy, the patient’s condition did not improve, with persistent symptoms prompting further evaluation.

At that point, the GeneXpert MTB/RIF (CEPHEID) test result from the bronchial lavage was received, detecting MTB in low quantity, with Rifampicin resistance being indeterminate. Although the test confirmed infection with Mtb, the low bacterial load not only prevented an accurate determination of Rifampicin resistance due to the insufficient number of bacilli but also necessitated further microbiological confirmation.

Despite this uncertainty, antituberculous therapy was initiated in accordance with national guidelines, adjusted to body weight, and included Isoniazid 300 mg/day, Rifampicin 600 mg/day, Ethambutol 1400 mg/day, and Pyrazinamide 1750 mg/day, all administered orally. Additionally, antihistamines were administered to prevent hypersensitivity reactions associated with antituberculous treatment, and intravenous corticosteroids (in tapering doses) were introduced to manage the right-sided pleural effusion.

During the first week of therapy, the patient experienced poor tolerance to treatment, presenting with nausea, vomiting, and diarrhea, which resolved following the administration of directly observed therapy (DOTS) at intervals and intravenous proton pump inhibitors (PPIs).

Repeated blood tests after one week showed a slight increase in transaminase levels, resolution of the inflammatory syndrome, and persistence of leukocytosis without monocytosis. The dynamic evolution of key laboratory parameters is summarized in Table 1.

After two weeks of hospitalization, the patient was discharged in a clinically stable cardiorespiratory condition, with instructions to continue antituberculous therapy under the supervision of the Territorial Dispensary and to undergo regular bacteriologic monitoring of sputum following national treatment protocols.

Drug susceptibility testing (DST) was conducted on BACTEC Liquid Culture, which returned a positive result after three weeks, confirming the presence of Mtb. Additionally, after two months, the Lowenstein-Jensen culture also tested positive, further validating the diagnosis. Both cultures demonstrated sensitivity to all first-line antituberculous drugs, with no evidence of drug resistance.

### 2.4. Follow-Ups

**Figure 3:**
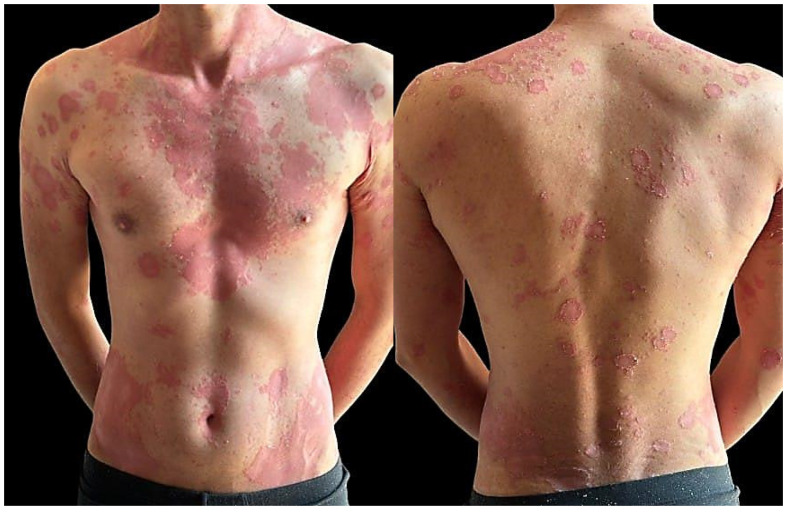
Clinical images taken two months after initiating antituberculous therapy, showing multiple well-demarcated, erythematous plaques with thick, silvery-white scales, localized on anterior chest and upper extremities, consistent with psoriasis vulgaris.

Two months after initiating therapy, the patient reported the gradual appearance of erythematous-squamous skin lesions, initially localized on the scalp, on the posterior thorax and chest, with progression to the limbs, suggesting systemic dermatologic involvement (Figure 3).

A dermatological examination revealed well-demarcated erythematous plaques covered with thick, whitish scales, revealing plaque psoriasis. A skin biopsy confirmed the diagnosis of hyperkeratosis, parakeratosis, and lymphohistiocytic inflammation and collections of neutrophils in the spinosum (spongiosiform pustules of Kogoj).

Given the diagnosis of vulgar psoriasis, the treating dermatologist initially recommended Apremilast, a phosphodiesterase-4 (PDE4) inhibitor. However, its initiation was contraindicated due to the ongoing antituberculous therapy, as immunomodulatory agents could impair the host’s capacity to respond to infection with Mtb. This highlights the need for a multidisciplinary approach to balance the management of comorbidities while minimizing risks.

As an alternative, Neotigason (Acitretin), a second-generation retinoid, was considered and administered at 10 mg twice daily for two months, accompanied by topical therapy with keratolytics ointments with salicylic acid, Hydrocortisone cream, Vaseline, and vitamin A. The patient exhibited a favorable clinical response, with remission of psoriatic lesions following treatment.

For TB management, monitoring was conducted through sputum examinations at months 2, 5, and 6 (T2, T5, T6), in accordance with the national protocol and under the supervision of the Territorial Dispensary. With two consecutive negative cultures, the patient was classified as a successfully treated TB case.

**Figure 4:**
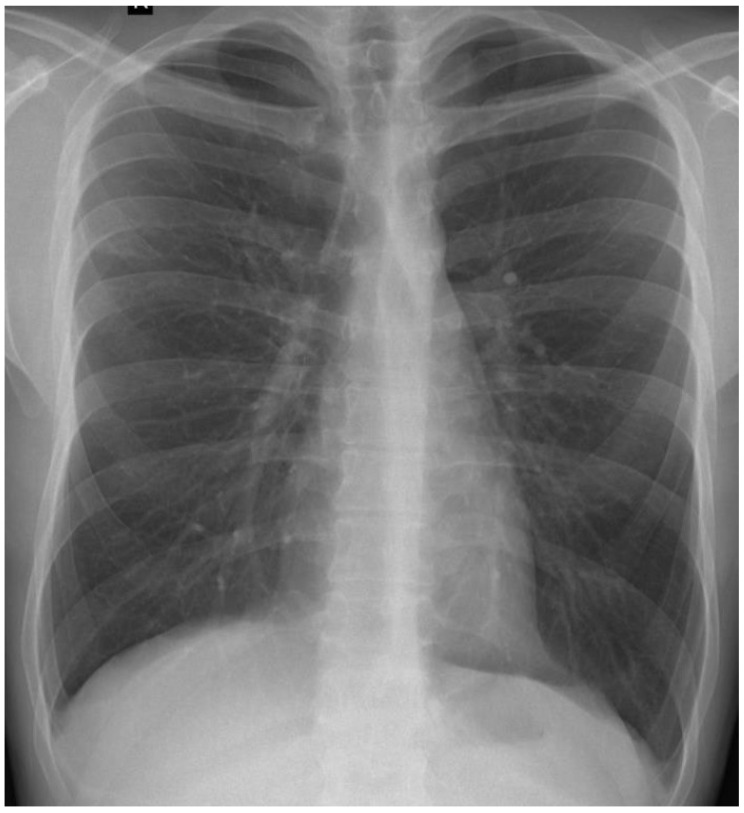
Chest X-ray at T6 shows complete resolution of right suprahilar opacity and no evidence of residual sequelae.

Serial chest radiographs over six months demonstrated a favorable radiological evolution, marked by the progressive resolution of pulmonary opacities, the absence of new infiltrative changes, and the lack of residual fibrotic or cavitary sequelae on follow-up imaging (Figure 4).

## 3. Discussions

This case highlights a rare and complex interplay between pulmonary TB and the subsequent development of vulgar psoriasis. The co-occurrence of these conditions, in the context of a family history of psoriatic arthritis and latent TB, raises important considerations regarding genetic predisposition, immune modulation, and treatment strategies.

Although psoriasis and TB are very different, there is an interesting parallel between the immune mechanisms involved in both diseases. T lymphocytes play a central role in both conditions, and proinflammatory cytokines such as TNF-α and IL-17 are involved [7].

### 3.1. The Role of TNF-α in TB and Psoriasis

TNF-α is an important cytokine in the pathogenesis of both psoriasis and TB [8]. In TB, TNF-α plays a dual role and is essential for macrophage activation to control infection and granuloma formation, [9,10]. According to Flynn and Chan, forming granulomas is critical in controlling the spread of Mtb and maintaining tissue integrity. Similarly, Olsen et al. emphasized the role of TNF-α in both acute and chronic infection phases, as evidenced by studies in animal models and patients receiving anti-TNF-α therapy [9,11]. However, in excessive amounts, TNF-α can also contribute to tissue destruction [12].

In psoriasis, TNF-α drives the inflammatory cascade, contributing to the skin lesions seen in affected individuals. The study, “Serum Levels of Tumor Necrosis Factor—Alpha in Patients with Psoriasis” by Ocvina-Kurtovic, Nermina, and Emina Kasumagic-Halilovic, reported that elevated serum levels of TNF-α were observed in psoriasis patients, demonstrating a strong correlation with disease severity. Furthermore, the authors highlighted TNF-α as a more reliable biomarker than other cytokines for monitoring psoriasis severity during follow-up [13].

### 3.2. IL-17: A Shared Cytokine in TB and Psoriasis

Cytokines like interleukin-17 (IL-17) and interleukin-23 (IL-23) contribute to keratinocyte hyperproliferation and skin inflammation. As discussed by Lowes and Krueger, IL-17 is one of the primary cytokines involved in psoriasis pathogenesis, promoting the recruitment of immune cells to the skin and amplifying the inflammatory process [14].

Interestingly, in TB, IL-17 is also implicated in the inflammatory environment in the lungs and supports granuloma formation [7]. Studies suggest that while IL-17 plays a protective role by recruiting neutrophils and enhancing granuloma integrity, excessive IL-17 production can lead to increased inflammation and tissue damage, particularly in the context of repeated BCG vaccination [15].

This highlights the delicate balance required for IL-17 regulation in TB, as its overproduction may exacerbate pathology rather than contribute to host defense.

### 3.3. CD4+ T Cells: A Central Component in Both Diseases

One of the similarities between TB and psoriasis is the activation of T cells, a central component of the immune response. In TB, CD4+ T cells are stimulated by mycobacterial antigens presented by infected macrophages, leading toproduction of interferon-gamma (IFN-γ). This cytokine enhances macrophage activation, promotes bacterial clearance, and facilitates granuloma formation, a critical mechanism for containing Mtb [10].

Similarly, in psoriasis, CD4+ T cells are activated by dendritic cells presenting self-antigens, releasing proinflammatory cytokines such as TNF-α, IL-17, and IL-22. These mediators sustain chronic inflammation, promote keratinocyte hyperproliferation, and drive the formation of psoriatic plaques, contributing to disease pathogenesis [16].

### 3.4. Shared Immune Mechanism and Clinical Implications

A noteworthy similarity between TB and psoriasis is the formation of immune cell aggregates, which, although differing in their exact form, share functional similarities. Conversely, immunosuppressive treatments for psoriasis, such as TNF inhibitors, increase the risk of TB reactivation, highlighting the need for careful management in patients with both conditions [17].

This cross-talk is an important consideration in managing patients with both conditions, as immunosuppressive therapies used in psoriasis may increase susceptibility to TB.

### 3.5. Comparative Discussions About the Case and the Literature Review

Several cases in the literature have highlighted the relationship between TB treatment and the development of psoriatic conditions. For instance, Sindhu’s “Psoriasiform Drug Eruption Induced by Anti-Tuberculosis Medication” describes psoriasiform eruptions linked to antituberculosis therapy [18], while Park et al. in “Psoriasiform Drug Eruption Induced by Anti-Tuberculosis Medication: Potential Role of Plasmacytoid Dendritic Cells” emphasized the involvement of plasmacytoid dendritic cells in similar drug-induced cases [19]. Both reports analyze the significant role of immune dysregulation triggered by antituberculosis medications in developing psoriatic-like eruptions.

Unlike these cases, however, our patient exhibits a notable genetic predisposition, evidenced by a family history of psoriatic arthritis. This genetic component has an important role in the pathogenesis of psoriasis in this context, potentially amplifying the immune response initiated by TB therapy.

Beyond genetic predisposition, one of the key elements supporting a link between TB and psoriasis in this case is the temporal association between the two conditions. The patient developed psoriatic lesions two months after the initiation of TB treatment, suggesting a possible immune-mediated response triggered either by the infection itself or by the host’s immune reaction to TB therapy. This aligns with existing reports in the literature describing psoriasis-like eruptions following TB treatment.

Moreover, the absence of other known psoriasis triggers strengthens the plausibility of a TB-induced mechanism. The patient had no prior history of psoriasis and was not exposed to common psoriasis triggers, such as streptococcal infections, beta-blockers, NSAIDs, or significant psychosocial stressors. This further suggests that the immune response to TB infection and/or its treatment played a pivotal role in triggering psoriasis in this case.

### 3.6. Therapeutic Considerations for Psoriasis in Active TB Infection

The patient was initially considered for treatment with Otezla (Apremilast), an oral medication for psoriasis, which works by reducing the production of proinflammatory cytokines, including TNF-α. Although clinical trials have not reported new cases of active tuberculosis linked to Apremilast, there have been instances of Mtb reactivation following its use in psoriasis treatment, indicating potential risks for certain vulnerable populations [20].

In the case of our patient, Apremilast was contraindicated due to its interaction with Rifampicin, a potent inducer of cytochrome P450 enzymes, which significantly reduces Apremilast’s therapeutic efficacy [21].

Given these challenges, topical therapy represents a first-line approach, offering a safe and effective alternative with minimal risk of interactions with antituberculous treatment. Current guidelines recognize keratolytics, including topical formulations with urea and salicylic acid, as adjunctive therapy for psoriasis. These treatments are internationally established as a standard of care for managing psoriasis of all severity levels [22]. In such cases, treatment can incorporate both the previously mentioned agents as well as a combination of Calcipotriol and Betamethasone ointment to enhance therapeutic efficacy [23].

For patients requiring systemic therapies, biological medications such as TNF-alpha inhibitors (Adalimumab, Etanercept, Infliximab), IL-12/IL-23 inhibitors (Ustekinumab), and IL-17 inhibitors (Secukinumab and Ixekizumab) are available for psoriasis treatment. However, TNF-alpha inhibitors must be used with extreme caution, as they can reactivate latent TB or exacerbate an active one, given the essential role of TNF-alpha in macrophage activation and granuloma formation, essential for infection control [9]. In contrast, Ustekinumab, Secukinumab, and Ixekizumab are generally considered safer alternatives, though they still require close monitoring due to infection risks. These agents should not be initiated during active TB but rather after the completion of chemoprophylaxis, under the supervision of a pulmonologist [24].

Furthermore, immunomodulatory agents such as Methotrexate can present significant risks, including hepatotoxicity, nephrotoxicity, exacerbation of active infections, and interactions with antituberculous treatments. For these reasons, these medications are not routinely recommended for psoriasis treatments in patients with active tuberculosis [25].

As an alternative systemic therapy, Acitretin, a second-generation retinoid, was selected due to its lack of immunosuppressive effects. Retinoids, both natural and synthetic, exhibit biological activity similar to vitamin A, which plays a crucial role in immune system modulation, cellular growth, differentiation, and proliferation [26].

Although Acitretin monotherapy is generally less effective for plaque psoriasis, it is well established in the treatment of generalized, palmoplantar pustular, and hyperkeratotic psoriasis variants. Additionally, Acitretin is particularly suitable for male patients, as it does not affect fertility, unlike in female patients where teratogenicity is a concern [27].

In this case, for our patient, the most effective and safest treatment recommended by the attending dermatologist was the initiation of oral Acicretin 20 mg per day for 2 months and the use of topical preparations with keratolytic substances, corticosteroids, and topical vitamin A, with total clearance of the lesions.

Our case highlights the crucial need for a multidisciplinary approach and individualized treatment strategies to ensure optimal disease control while minimizing risks. The management of a patient with both active tuberculosis and moderate-to-severe psoriasis requires close collaboration between dermatologists and pulmonologists, allowing for careful selection of therapeutic options that address both conditions without compromising immune function or increasing the risk of TB reactivation.

Furthermore, treatment decisions must consider not only disease severity but also potential drug interactions, adverse effects, and long-term patient outcomes. This case reinforces the importance of a personalized, patient-centered approach, ensuring that therapeutic interventions are both clinically effective and immunologically safe in complex comorbid conditions.

## 4. Conclusions

This case underscores the rare coexistence of active pulmonary Tb and vulgar psoriasis, with complex interactions between infectious and autoimmune diseases. The significant genetic predisposition in this patient, evidenced by a family history of psoriatic arthritis, emphasizes the role of genetics in pathogenesis.

The shared immunological pathways, particularly those involving TNF-α, IL-17, and CD4+ T cells, illustrate the delicate balance required in overlapping conditions. Further research is needed to better understand the interaction between genetic susceptibility and immune response in such cases, potentially guiding more personalized and effective treatment strategies.

Taken together, these findings support the hypothesis that TB acted as an environmental trigger for psoriasis development in a genetically predisposed individual, highlighting the complex interplay among infectious diseases, immune regulation, and autoimmune disorders.

## Figures and Tables

**Table 1:** Blood test results in dynamics: admission versus after one week of hospitalization.

Parameter	On Admission	After 1 Week	Normal Values
AST	16 U/L	44 U/L	3–40 U/L
ALT	23 U/L	31 U/L	3–41 U/L
Erythrocyte Sedimentation Rate	25 mm/30 min	10 mm/30 min	0–15 mm/30 min
Uric acid	2.7 mg/dL	4.25 mg/dL	3.5–7.00 mg/dL
Fibrinogen	4.55 g/L	2.75 g/L	2.00–4.00 g/L
Leukocytes	10.84 × 10^3^/μL	11.25 × 10^3^/μL	4–10 × 10^3^/μL
Monocytes	1.47 × 10^3^/μL	0.85 × 10^3^/μL	0–1 × 10^3^/μL

## Data Availability

All data presented in this report are included in the article. Further inquiries can be directed to the corresponding author.

## References

[1] Villar-Hernández R., Ghodousi A., Konstantynovska O., Duarte R., Lange C., Raviglione M., on behalf of the UNITE4TB Consortium (2023). Tuberculosis: Current Challenges and Beyond. Breathe.

[2] Gill C.M., Dolan L., Piggott L.M., McLaughlin A.M. (2022). New Developments in Tuberculosis Diagnosis and Treatment. Breathe.

[3] Duffin K.C., Chandran V., Gladman D.D., Krueger G.G., Elder J.T., Rahman P. (2008). Genetics of psoriasis and psoriatic arthritis: Update and future direction. J. Rheumatol..

[4] Raharja A., Mahil S.K., Barker J.N. (2021). Psoriasis: A Brief Overview. Clin. Med..

[5] World Health Organization (2016). Global Report on Psoriasis.

[6] Megna M., Patruno C., Bongiorno M.R., Gambardella A., Guarneri C., Foti C., Lembo S., Loconsole F., Fabbrocini G. (2022). Lack of Reactivation of Tuberculosis in Patients with Psoriasis Treated with Secukinumab in a Real-World Setting of Latent Tuberculosis Infection. J. Dermatol. Treat..

[7] Sarkar S., Panda S., Kim B., Raychaudhuri S.K., Ghosh A., Raychaudhuri S.P. (2020). Risk of Tuberculosis with Anti-Tumor Necrosis Factor-Alpha Therapy in Patients with Psoriasis and Psoriatic Arthritis in Indian Population. Indian J. Dermatol. Venereol. Leprol..

[8] Kasiraman V., Atwan A.A., Durojaiye O.C., Kalavala M., Piguet V. (2014). Risk of Tuberculosis with the Use of Anti-TNF Medications in Psoriasis: Incidence, Screening and Management. Dermatol. Online J..

[9] Mastorino L., Dapavo P., Trunfio M., Avallone G., Rubatto M., Calcagno A., Ribero S., Quaglino P. (2022). Risk of Reactivation of Latent Tuberculosis in Psoriasis Patients on Biologic Therapies: A Retrospective Cohort from a Tertiary Care Centre in Northern Italy. Acta Derm. Venereol..

[10] Sia J.K., Rengarajan J. (2001). Immunology of Tuberculosis. Annu. Rev. Immunol..

[11] Olsen A., Chen Y., Ji Q., Zhu G., De Silva A.D., Vilchèze C., Weisbrod T., Li W., Xu J., Larsen M. (2016). Targeting Mycobacterium Tuberculosis Tumor Necrosis Factor Alpha-Downregulating Genes for the Development of Antituberculous Vaccines. mBio.

[12] Society for Investigative Dermatology (2017). Society for Investigative Dermatology 2016 Board of Directors Meeting Minutes. J. Investig. Dermatol..

[13] Ovcina-Kurtovic N., Kasumagic-Halilovic E. (2022). Serum Levels of Tumor Necrosis Factor—Alpha in Patients with Psoriasis. Mater. Socio-Medica.

[14] Lowes M.A., Bowcock A.M., Krueger J.G. (2007). Pathogenesis and therapy of psoriasis. Nature.

[15] Cruz A., Fraga A.G., Fountain J.J., Rangel-Moreno J., Torrado E., Saraiva M., Pereira D.R., Randall T.D., Pedrosa J., Cooper A.M. (2010). Pathological Role of Interleukin 17 in Mice Subjected to Repeated BCG Vaccination after Infection with Mycobacterium Tuberculosis. J. Exp. Med..

[16] Jariwala S.P. (2007). The Role of Dendritic Cells in the Immunopathogenesis of Psoriasis. Arch. Dermatol. Res..

[17] Gudjonsson J.E., Johnston A., Sigmundsdottir H., Valdimarsson H. (2004). Immunopathogenic Mechanisms in Psoriasis. Clin. Exp. Immunol..

[18] Sindhu N. (2023). PSORIASIFORM DRUG ERUPTION INDUCED BY ANTI-TUBERCULOSIS MEDICATION: A CASE REPORT. Int. J. Curr. Pharm. Res..

[19] Park J.J., Choi Y.D., Lee J.B., Kim S.J., Lee S.C., Won Y.H., Yun S.J. (2010). Psoriasiform drug eruption induced by anti-tuberculosis medication: Potential role of plasma-cytoid dendritic cells. Acta Derm. Venereol..

[20] Adams L., Smith E.L., Tilakaratne D., Krause V. (2024). Tuberculosis Reactivation Following Apremilast Therapy for Psoriasis: Time to Consider Routine TB Screening?. Australas. J. Dermatol..

[21] European Medicines Agency Otezla: EPAR—Product Information. https://www.ema.europa.eu/en/documents/product-information/otezla-epar-product-information_en.pdf.

[22] Nast A., Boehncke W.H., Mrowietz U., Ockenfels H.M., Philipp S., Reich K., Rosenbach T., Sammain A., Schlaeger M., Sebastian M. (2012). German S3-Guidelines on the Treatment of Psoriasis Vulgaris (Short Version). Arch. Dermatol. Res..

[23] Megna M., Lauletta G., Tommasino N., Salsano A., Battista T., Ruggiero A., Martora F., Potestio L. (2024). Management of Psoriasis Patients with Serious Infectious Diseases. Adv. Ther..

[24] Abdulwahhab W.S., Mehair A.S., Iedi A.S.F. (2022). Ustekinumab Treatment in Patients with Moderateto-Severe Psoriasis and Latent Tuberculosis Infection: A Study of 3 Case Reports. J. Cosmet. Dermatol. Sci. Appl..

[25] Richard, Warren B., Griffiths C.E.M. (2008). Systemic therapies for psoriasis: Methotrexate, retinoids, and cyclosporine. Clin. Dermatol..

[26] Zito P.M., Patel P., Mazzoni T. (2025). Acitretin. StatPearls.

[27] Akarsu S. (2023). How should we do in the selection and follow-up of systemic conventional treatments in psoriasis?. Explor. Musculoskelet. Dis..

